# Intra- and Extracellular Pillars of a Unifying Framework for Homeostatic Plasticity: A Crosstalk Between Metabotropic Receptors and Extracellular Matrix

**DOI:** 10.3389/fncel.2019.00513

**Published:** 2019-11-19

**Authors:** Lorenzo A. Cingolani, Carmela Vitale, Alexander Dityatev

**Affiliations:** ^1^Department of Life Sciences, University of Trieste, Trieste, Italy; ^2^Center for Synaptic Neuroscience and Technology (NSYN), Istituto Italiano di Tecnologia, Genoa, Italy; ^3^Department of Experimental Medicine, University of Genoa, Genoa, Italy; ^4^Molecular Neuroplasticity, German Center for Neurodegenerative Diseases (DZNE), Magdeburg, Germany; ^5^Medical Faculty, Otto von Guericke University Magdeburg, Magdeburg, Germany; ^6^Center for Behavioral Brain Sciences, Magdeburg, Germany

**Keywords:** mGluRs, extracellular matrix, HCN channels, SK channels, AMPARs, ADAMTS, dopamine receptors, 5-HT7 receptors

## Abstract

In the face of chronic changes in incoming sensory inputs, neuronal networks are capable of maintaining stable conditions of electrical activity over prolonged periods of time by adjusting synaptic strength, to amplify or dampen incoming inputs [homeostatic synaptic plasticity (HSP)], or by altering the intrinsic excitability of individual neurons [homeostatic intrinsic plasticity (HIP)]. Emerging evidence suggests a synergistic interplay between extracellular matrix (ECM) and metabotropic receptors in both forms of homeostatic plasticity. Activation of dopaminergic, serotonergic, or glutamate metabotropic receptors stimulates intracellular signaling through calmodulin-dependent protein kinase II, protein kinase A, protein kinase C, and inositol trisphosphate receptors, and induces changes in expression of ECM molecules and proteolysis of both ECM molecules (lecticans) and ECM receptors (NPR, CD44). The resulting remodeling of perisynaptic and synaptic ECM provides permissive conditions for HSP and plays an instructive role by recruiting additional signaling cascades, such as those through metabotropic glutamate receptors and integrins. The superimposition of all these signaling events determines intracellular and diffusional trafficking of ionotropic glutamate receptors, resulting in HSP and modulation of conditions for inducing Hebbian synaptic plasticity (i.e., metaplasticity). It also controls cell-surface delivery and activity of voltage- and Ca^2+^-gated ion channels, resulting in HIP. These mechanisms may modify epileptogenesis and become a target for therapeutic interventions.

## Introduction

Homeostatic plasticity enables neurons to stabilize network activity within an optimal dynamic range over prolonged periods of time, thereby playing a fundamental neuroprotective role during pathological conditions that tend to alter function and integrity of neuronal networks. Homeostatic plasticity entails negative feedback mechanisms that can alter diverse aspects of network function: the number of shared connections, the strength of excitatory and inhibitory synaptic transmission, the excitatory/inhibitory ratio [phenomena collectively designated as homeostatic synaptic plasticity (HSP)] and the level of intrinsic excitability [homeostatic intrinsic plasticity (HIP)] (Silberberg et al., 2005; Hahn et al., 2019). Recent evidence suggests that many of these homeostatic mechanisms are not always active but instead are triggered by behavioral states, such the sleep–wake rhythm, and by modulatory neurotransmitters and metabotropic receptors, such as the glutamatergic, dopaminergic, and serotonergic receptors (Tononi and Cirelli, 2014; Hengen et al., 2016; Diering et al., 2017).

In addition to extensive analyses of ion channel trafficking and intracellular signaling pathways involved in the different forms of homeostatic plasticity, several studies have revealed the importance of synaptic extracellular matrix (ECM) molecules, such as neuronal activity-regulated pentraxin (Narp) (Chang et al., 2010), and major ECM receptors such as β3 integrin (Cingolani et al., 2008; McGeachie et al., 2012). More recently, the attention was drawn also to the hyaluronic-acid-based perisynaptic ECM (Korotchenko et al., 2014; Valenzuela et al., 2014), incorporating lecticans, link proteins, and tenascin-R (Ferrer-Ferrer and Dityatev, 2018). Here, we review emerging common themes linking ECM remodeling with other major mechanisms of homeostatic plasticity, which are intriguingly “clustered” around regulation of metabotropic receptors.

## mGluRs in Homeostatic Synaptic Plasticity

Metabotropic glutamate receptors (mGluRs) represent a prominent family of class C G protein-coupled receptors (GPCRs). These receptors assemble into constitutive dimers with each subunit comprising a “Venus flytrap” domain, a large extracellular N-terminal domain that contains the endogenous ligand-binding site (Pin and Bettler, 2016). Based on sequence homology, G-protein coupling, and ligand selectivity, we can distinguish three major groups of mGluRs. In neurons, group I mGluRs (mGluR1 and 5) are enriched in postsynaptic compartments where they couple to Gα_q_ heterotrimeric G proteins and activate phospholipase C. Group II (mGluR2 and 3) and III mGluRs (mGluR4, 6, 7, and 8) are instead localized mainly presynaptically where they couple to Gα_i/o_ and inhibit adenylyl cyclase (Niswender and Conn, 2010).

**FIGURE 1:**
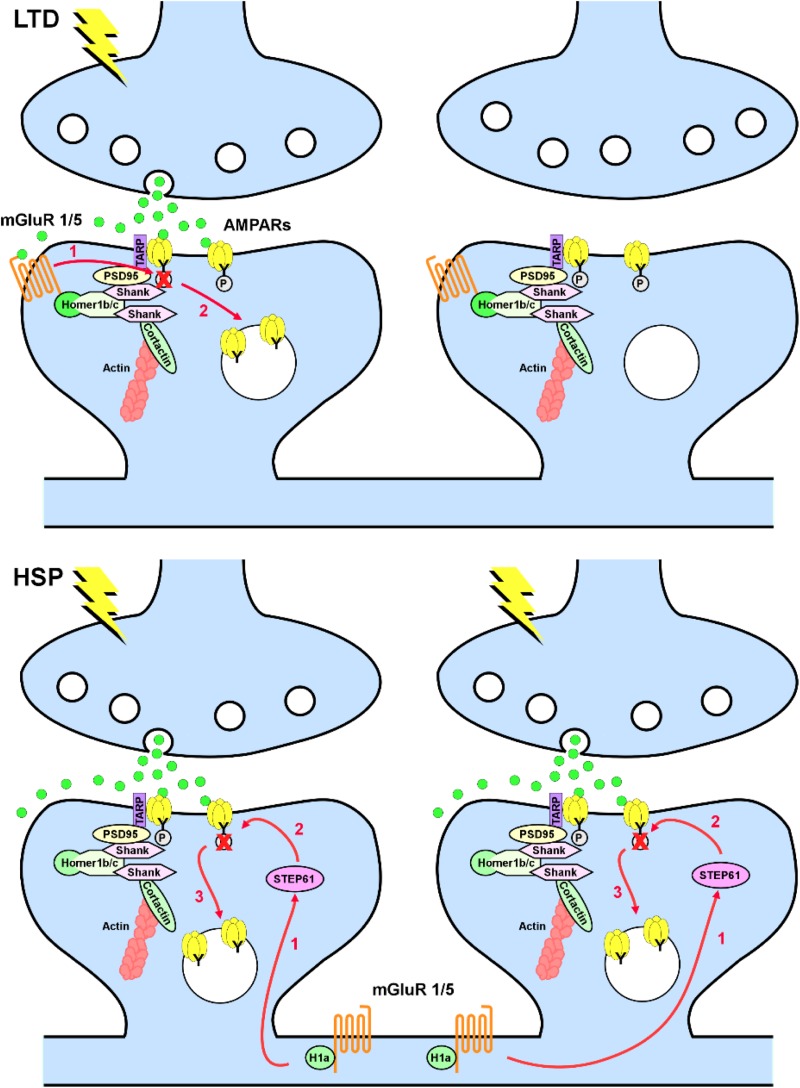
Metabotropic glutamate receptors 1/5 (mGluR1/5) in long-term depression (LTD) and homeostatic synaptic plasticity (HSP). Top, in LTD, mGluR1/5 are anchored at perisynaptic sites via Homer 1b/c and activated in a synapse-specific manner by synaptically released glutamate. Activation of mGluR1/5 leads to tyrosine dephosphorylation of the GluA2 subunit of AMPARs (1), with consequent increase in AMPAR endocytosis (2). Bottom, in HSP induced by chronic increase in network activity, induction of Homer1a decouples mGluR1/5 from the synaptic signaling machinery and induce a constitutive glutamate-independent activation of mGluR1/5. Homer1a-induced mGluR1/5 signaling requires upregulation of the striatal-enriched protein tyrosine phosphatase (STEP61; 1), with consequent dephosphorylation of the GluA2 subunit of AMPARs (2) and increase in AMPAR endocytosis (3) in a non-synapse-specific manner.

Group I mGluRs are involved in the induction of both Hebbian and homeostatic forms of synaptic plasticity. The mechanism of activation of these receptors in the two forms of plasticity is, however, different. In Hebbian mGluR-induced long-term depression (mGluR-LTD), mGluR1/5 are activated by synaptically released glutamate; consequently, only mGluR1/5 localized in close proximity to the activated synapses will contribute to weakening of synaptic transmission in a synapse-specific manner (Oliet et al., 1997; Luscher and Huber, 2010). Conversely, in homeostatic synaptic downscaling, mGluR1/5 are activated by the immediate early gene Homer1a, which is induced in response to the increase in network activity. Rather than being synapse specific, Homer1a induction is cell wide and promotes mGluR1/5 activity in a glutamate-independent manner by disrupting the scaffold formed by the constitutively expressed long forms of Homer, which firmly anchor mGluR1/5 at perisynaptic sites (Ango et al., 2001; Hu et al., 2010). Because disruption of mGluR1/5 clusters favors constitutive activation of these receptors, Homer1a acts effectively as an endogenous mGluR1/5 allosteric modulator. It is noteworthy that, albeit different in the induction mechanism, mGluR-LTD and homeostatic synaptic downscaling eventually converge as both forms of synaptic plasticity induce tyrosine dephosphorylation of GluA2 subunits of AMPA-type glutamate receptors (AMPARs), with a consequent increase in the internalization rate of GluA2-containing AMPARs (Figure 1; Moult et al., 2006; Gladding et al., 2009; Scholz et al., 2010; Jang et al., 2015). Interestingly, these mechanisms appear to be relevant to synapse remodeling and memory consolidation during sleep because the synaptic levels of Homer1a are dramatically increased during sleep, leading to loss of synaptic mGluR5, constitutive activation of these receptors, and weakening of synapses (Diering et al., 2017).

## Reciprocal Interactions Between Extracellular Environment and mGluRs in Homeostatic Synaptic Plasticity

Functional characterization of mGluRs has focused predominantly on proteins involved in intracellular scaffolding and signaling (O’Connor et al., 2014). However, it is becoming increasingly clear that mGluRs also associate with cell adhesion molecules (CAMs) and ECM components and that these interactions play a crucial role in regulating localization and signaling of mGluRs. Recently, group III mGluRs have been shown to interact with ELFN1 [extracellular leucine-rich repeat (LRR) and fibronectin type III domain-containing 1 (Tomioka et al., 2014; Cao et al., 2015; Wang et al., 2017; Dunn et al., 2018)], a member of the family of LRR CAMs, which play an essential role in specifying synaptic connectivity (de Wit and Ghosh, 2016). The interaction likely involves the glutamate-binding domains on mGluRs and the N-terminal LRR protein-interaction domain on ELFN1 (Stachniak et al., 2019). In the hippocampus and cortex, ELFN1 is found exclusively in somatostatin interneurons from where it interacts transsynaptically with presynaptic mGluR7 expressed in pyramidal neurons, thereby recruiting mGluR7 selectively at synapses between pyramidal neurons and somatostatin interneurons (Tomioka et al., 2014). The enrichment of mGluR7 is responsible for reducing neurotransmitter release probability and for endowing these synapses with their distinctive short-term facilitation properties (Sylwestrak and Ghosh, 2012). Similarly, in the retina, transsynaptic interaction between ELFN1 and mGluR6 plays an essential role in retaining mGluR6 at the synapses between rods and bipolar cells (Cao et al., 2015; Wang et al., 2017). These observations exemplify the relevance of extracellular interactions for clustering mGluRs at synapses.

Perhaps more importantly, recent work suggests that ELFN1 has not only a structural role, but it could also promote constitutive activation of group III mGluRs. Specifically, the interaction between ELFN1 and group III mGluRs may favor dimerization of these receptors and stabilize them in a constitutive active conformation (Dunn et al., 2018; Stachniak et al., 2019). In this model, postsynaptic ELFN1 would act therefore as an endogenous allosteric modulator to bias group III mGluR activity from a glutamate-induced to a tonic-signaling mode. This dual role of ELFN1 as scaffold protein and allosteric modulator is closely reminiscent of the well-characterized function of Homer proteins in regulating localization and basal activity of group I mGluRs in homeostatic plasticity (Ango et al., 2001; Hu et al., 2010; Shim et al., 2016). Crucially, the interplay between ELFN1 and mGluR7 is physiologically relevant because loss of either proteins induces similar phenotypes in mice, specifically hyperactivity and increased susceptibility to seizures (Sansig et al., 2001; Dolan and Mitchell, 2013; Tomioka et al., 2014).

The interplay between ECM and mGluRs is twofold: on the one hand, the extracellular environment controls mGluRs, as exemplified above, but on the other hand, the signaling through mGluRs modulates the extracellular environment. For instance, stimulation of group I mGluRs activates the disintegrin metalloproteinase tumor necrosis factor-α-converting enzyme (TACE; alias, ADAM 17), which in turn cleaves the membrane protein neuronal pentraxin receptor (NPR). This process, known as “shedding,” induces the release of a soluble ectodomain of NPR, which coclusters the pentraxin Narp and AMPARs through extracellular interactions, and stimulates AMPAR endocytosis. Remarkably, this mechanism is relevant for both hippocampal and cerebellar mGluR-LTD, which rely otherwise on divergent signaling pathways (Cho et al., 2008).

Although it is not known whether similar signaling pathways are engaged in homeostatic plasticity, it is worth noting that one of the best-studied substrates of TACE is tumor necrosis factor alpha (TNF-α), which is required for inactivity-induced HSP both *in vitro* and *in vivo* (Stellwagen and Malenka, 2006; Kaneko et al., 2008). TNF-α increases surface expression of β3 integrin, which interacts directly with the GluA2 subunit of AMPARs and is required for regulating network activity and HSP but not mGluR-LTD (Cingolani et al., 2008; McGeachie et al., 2012; Pozo et al., 2012; Jaudon et al., 2019). In addition, under conditions of hyperactivity, expression and secretion of the pentraxin Narp is rapidly and dramatically upregulated, which promotes clustering and retention of AMPARs on parvalbumin-expressing interneurons, thus increasing excitatory inputs to these cells, which culminates in homeostatic upregulation of principal cell inhibition (Chang et al., 2010). Accordingly, Narp^–/–^ mice display increased sensitivity to kindling-induced seizures.

## Metabotropic Receptor-Driven Ecm Remodeling and Homeostatic Synaptic Plasticity

Like TACE-induced extracellular proteolysis is important for downregulation of excitatory transmission, disintegrin and metalloprotease with thrombospondin motifs (ADAMTS)-mediated proteolytic modifications of ECM are associated with inactivity-induced homeostatic synaptic upscaling (Valenzuela et al., 2014). Using an antibody specific for a brevican fragment cleaved by the matrix metalloproteases ADAMTS4 and 5, the researchers revealed perisynaptic brevican processing by these proteases. Interestingly, after induction of homeostatic plasticity in neuronal cell cultures by prolonged network inactivity, there is an increased brevican processing at inhibitory as well as excitatory synapses, corresponding to the ADAMTS4 subcellular localization. This study suggests therefore a permissive role of perisynaptic ECM remodeling in removing inhibitory constrains of synaptic growth necessary for synaptic upscaling.

Which factors control the activity of ADAMTS and other extracellular proteases and hence the integrity of perisynaptic ECM? Recent findings implicate dopaminergic and serotonergic neuromodulation. Activation of D1-type dopamine (DA) receptors induces proteolysis of brevican and aggrecan via ADAMTS4 and 5 specifically at excitatory synapses of rat cortical neurons (Mitlöhner et al., 2019). Pharmacological inhibition and short hairpin RNA-mediated knockdown of ADAMTS4 and 5 reduces brevican cleavage. The study further demonstrates that synaptic activity and DA neuromodulation are linked to ECM rearrangements via increased cAMP levels, NMDA receptor (NMDAR) activation, and signaling via protein kinase A (PKA) and the Ca^2+^/calmodulin-dependent protein kinase II (CaMKII). These findings are in line with the previously reported increase in the extracellular activity of the tissue plasminogen activator (tPA) protease after activation of D1-like DA receptors via a PKA-dependent pathway (Ito et al., 2007). Strikingly, tPA may directly activate ADAMTS4 (Lemarchant et al., 2014), suggesting that at least partially elevated remodeling of perisynaptic ECM may be due to tPA-ADAMTS4 processing. Previous analysis of tPA function in homeostatic plasticity had revealed a bidirectional effect of tPA on the composition of the postsynaptic density (PSD) (Jeanneret and Yepes, 2017). In inactive neurons, tPA induces phosphorylation and accumulation of pCaMKIIα in the PSD, resulting in pCaMKIIα-induced phosphorylation and synaptic recruitment of GluA1-containing AMPARs. In active neurons, tPA drives pCaMKIIα and pGluA1 dephosphorylation and subsequent removal from the PSD. These effects require active NMDARs and cyclin-dependent kinase 5 (Cdk5)-induced phosphorylation of the protein phosphatase 1 (PP1). Thus, tPA, and hence ADAMTS4 and potentially other members of the ADAMTS family, may act as homeostatic regulators of the postsynaptic efficacy in a CaMKII-dependent manner. In addition, enzymatic digestion of highly sulfated forms of heparan sulfates with heparinase I was reported to induce homeostatic synaptic upscaling in association with upregulated phosphorylation of CaMKII in cultured hippocampal neurons (Korotchenko et al., 2014). This is noteworthy, as heparan sulfate proteoglycans are major components of the ECM and play key roles in misfolding, oligomerization, and fibrillation of amyloidogenic proteins, stabilization of protein aggregates, as well as for cellular uptake of proteopathic seeds during their spreading (Maiza et al., 2018).

In contrast to DA, serotonin (5-HT) induces ECM remodeling by activating the matrix metalloproteinase 9 (Bijata et al., 2017). This study revealed a physical interaction between 5-HT7 receptors and CD44, the major receptor of the neural ECM backbone, hyaluronic acid. 5-HT7 receptor stimulation increases local matrix metalloproteinase 9 activity, which leads to CD44 cleavage and Cdc42 activation, followed by an increase in neuronal outgrowth and elongation of dendritic spines. Although there is no experimental evidence that this signaling may induce homeostatic plasticity, hyaluronic acid is known to control activity of postsynaptic L-type Ca^2+^ channels (Kochlamazashvili et al., 2010), which have been implicated in inactivity-induced HSP (Thiagarajan et al., 2005). Indeed, enzymatic digestion of hyaluronic acid leads to epileptiform activity *in vitro* (Vedunova et al., 2013), and mice deficient in hyaluronic acid synthase HAS3 show epileptic seizures (Arranz et al., 2014).

## mGluRs in Homeostatic Intrinsic Plasticity

Homeostatic adaptation of neuronal firing following prolonged changes in sensory inputs can be achieved not only by adjusting synaptic strength, to amplify or dampen incoming inputs (i.e., HSP), but also by altering intrinsic excitability (i.e., HIP). Observed initially in primary cortical cultures in response to the same pharmacological manipulations that induce HSP (Desai et al., 1999), HIP has been shown to contribute to network stability of various brain regions *in vivo*, often in cooperation with HSP (Debanne et al., 2019). As for HSP, both sensory deprivation and elevated network activity, as observed in status epilepticus, can induce HIP (Maffei and Turrigiano, 2008; Kirchheim et al., 2013; Kuba et al., 2015; Milshtein-Parush et al., 2017). Although the molecular mechanisms and ion channels that contribute to stabilizing intrinsic excitability vary widely according to the brain region and neuron type considered, much attention has been given to hyperpolarization-activated, cyclic nucleotide-gated (HCN) and K^+^ channels. Here, we will consider the contribution of HCN channels and of a subclass of K^+^ channels, the small-conductance Ca^2+^-activated K^+^ channels (SK channels) to HIP, and their interplay with metabotropic signaling and ECM.

### HCN Channels

Hyperpolarization-activated, cyclic nucleotide-gated channels, whose family comprises four members (HCN1, 2, 3, and 4), are of special interest because they are activated by membrane hyperpolarization, but they mediate a mixed Na^+^ and K^+^ current (I_h_), whose net effect is depolarizing. This means that opening (and closing) of HCN channels will counteract membrane hyperpolarization (and depolarization), thereby stabilizing membrane potential. Crucially, this negative-feedback regulation occurs also in the subthreshold range because HCN channels are partially open at voltages near the resting membrane potential (Biel et al., 2009). HCN channels play also a key role in regulating dendritic integration in CA1 hippocampal and layer V cortical pyramidal neurons. In these neurons, the dendritic density of HCN channels, and most notably of HCN1, increases dramatically along the apical dendrites with distance from the soma. As a consequence of this somato-dendritic gradient, HCN1 effectively dampens excitatory synaptic currents originating in distal apical dendrites, thus limiting their temporal summation (Stuart and Spruston, 2015).

A complex network of cell-autonomous, non-cell-autonomous, and activity-dependent mechanisms regulates distal dendritic targeting of HCN1 in pyramidal neurons. For example, the brain-specific HCN channel auxiliary subunit tetratricopeptide repeat-containing Rab8b-interacting protein (TRIP8b) supports dendritic enrichment of HCN1 via intracellular interactions (Piskorowski et al., 2011). The ECM protein Reelin provides instead a non-cell-autonomous extracellular factor for anchoring HCN1 at distal dendrites. Reelin is a large glycoprotein whose signaling is important for regulating both neuronal positioning during development and synaptic plasticity in the adult brain (Ferrer-Ferrer and Dityatev, 2018). In the adult, it is secreted by a subset of inhibitory interneurons with a non-uniform distribution across the hippocampus and the neocortex. This sets the conditions for establishing gradients of Reelin across these two brain regions. Binding of Reelin to the lipoprotein receptors, the apolipoprotein E receptor type 2 (APOER2) and the very low-density lipoprotein receptor (VLDLR), on pyramidal neurons activates Src family tyrosine kinases and the cytoplasmic signaling molecule Dab1. This signaling pathway promotes Hebbian synaptic plasticity by tyrosine phosphorylation of NMDARs (Beffert et al., 2005) and is required for giving the distal dendritic compartments of CA1 and layer V pyramidal neurons their molecular identity, including the enrichment in HCN1 (Kupferman et al., 2014).

Hyperpolarization-activated, cyclic nucleotide-gated channel expression is also under the control of neuronal activity both *in vitro* (Brager and Johnston, 2007; Shin and Chetkovich, 2007; Arimitsu et al., 2009; Gasselin et al., 2015; Shim et al., 2016; Schanzenbacher et al., 2018) and *in vivo*. Indeed, whisker trimming, to induce sensory deprivation in the barrel cortex, causes a decrease in HCN channel density in the distal region of the apical dendrites of layer V pyramidal neurons (Breton and Stuart, 2009). The network-activity-dependent regulation is bidirectional as pharmacological treatments that increase and decrease network activity up- and downregulate HCN activity, respectively. These adaptations are homeostatic because HCN channels actively oppose excitatory drive. Interestingly, they also play an essential metaplastic role as they counterbalance the complementary changes in synaptic strength that take place following HSP, thus ensuring that the propensity to induce Hebbian long-term potentiation (LTP) does not vary following chronic changes in network activity (Gasselin et al., 2015).

In hippocampal CA1 pyramidal neurons, more proximal apical dendrites are innervated by the Schaffer collateral pathway from CA3 pyramidal neurons, while distal apical dendrites are contacted by the perforant pathway from the entorhinal cortex (Megias et al., 2001). The localization of HCN1 in distal dendrites of CA1 neurons requires the activity of the perforant pathway and opening of NMDARs with subsequent elevation of intracellular Ca^2+^ and activation of CaMKII (Shin and Chetkovich, 2007). Conversely, activation of group I mGluRs at the Schaffer collateral and downstream activation of PKC downregulates HCN channels (Brager and Johnston, 2007). It is therefore plausible that a differential balance between NMDAR and mGluR1/5 signaling at the Schaffer collateral and perforant pathway synapses (Xu et al., 2010) may contribute to distal HCN enrichment.

As opposed to the situation in pyramidal neurons, HCN channels are uniformly distributed on the dendrites of cerebellar Purkinje cells (Angelo et al., 2007). Furthermore, neuronal activity affects the expression of HCN channels in Purkinje cells and pyramidal neurons in opposite directions. In Purkinje cells, chronic activity deprivation upregulates, rather than downregulating, HCN channels, thus decreasing the excitability of these neurons. Because Purkinje cells are inhibitory, these adaptations have a net homeostatic effect on network function. It is worth noting that HIP in Purkinje cells is initiated by glutamate-independent activation of mGluR1 (Shim et al., 2016), similarly to what happens for HSP in cortical neurons (Hu et al., 2010). Hence, constitutive group I mGluR signaling is important for the induction of both HSP and HIP and may change dramatically neuronal network function and stability. For example, in CA3 hippocampal pyramidal neurons, transient pharmacological stimulation of group I mGluRs appears to switch mGluR1 into a constitutive active state with consequent changes in multiple intrinsic ion conductances [including suppression of the Ca^2+^-dependent K^+^ current mediating the slow afterhyperpolarization (sI_AHP_) and activation of a voltage-gated cationic, TRPC-like current (I_mGluR(V)_)], which have an overall epileptogenic effect in the hippocampus (Bianchi et al., 2009, 2012; Young et al., 2013).

### SK Channels

Small-conductance Ca^2+^-activated K^+^ channels, whose family comprises four members (SK1–4), are voltage-independent K^+^ channels broadly expressed in the brain (Stocker and Pedarzani, 2000; Pedarzani and Stocker, 2008; Gymnopoulos et al., 2014). Low concentrations (in the submicromolar range) of intracellular Ca^2+^ activate SK channels by binding to calmodulin, which serves as intrinsic Ca^2+^ sensing subunit. In addition to calmodulin, SK channels interact constitutively with protein kinase CK2 and protein phosphatase 2A, which modulate Ca^2+^ sensitivity (Adelman et al., 2012). Because SK channels hyperpolarize membrane potential in response to intracellular Ca^2+^ rises, they have a well-recognized role in counteracting somatic excitability and Hebbian synaptic plasticity (Lujan et al., 2009). Recent evidence suggests a possible role for SK channels also in homeostatic plasticity. Notably, SK2 channels colocalize and coassemble with mGluR1 and mGluR5 in Purkinje cells and hippocampal pyramidal neurons, respectively (Garcia-Negredo et al., 2014; Lujan et al., 2018). In CA1 hippocampal and layer V cortical pyramidal neurons, stimulation of group I mGluRs activates inositol trisphosphate receptors (IP_3_Rs), which support intracellular Ca^2+^ waves in dendrites and somata. While these Ca^2+^ waves often evoke a transient SK-mediated hyperpolarization (Hagenston et al., 2008; El-Hassar et al., 2011), selective pharmacological stimulation of mGluR5 reduces SK currents in layer V pyramidal neurons (Sourdet et al., 2003; Cannady et al., 2017).

Our recent data indicate that the ECM proteoglycan brevican may constitutively inhibit activity of group III mGluRs postsynaptically in CA1 pyramidal neurons (Song et al., 2019). Under conditions of brevican deficiency, these receptors, however, become active and reduce cAMP levels in neurons. This results in inhibition of PKA activity, which normally drives endocytosis of SK channels, and hence in increased cell surface expression of SK channels and reduced excitability of pyramidal neurons. Such mechanism may be induced by activity-dependent proteolysis of brevican and plays therefore a homeostatic role by reducing dendritic neuronal excitability.

## Heparan Sulfate Proteoglycans in Axonal Excitability

Similar to dendritic, also axonal excitability is under the control of ECM molecules, which accumulate at the axon initial segment (AIS). Among these molecules are tenascin-R and heparan sulfate proteoglycans of glypican and syndecan subfamilies. Acute treatment of hippocampal slices with heparinase I results in impaired LTP due to a reduction in axonal excitability (Minge et al., 2017). Our recent findings demonstrate elevated CaMKII activity 24 h after intrahippocampal heparinase I injection *in vivo*, which is accompanied by reduced axonal excitability and impaired context discrimination in fear conditioning paradigms (Mironov et al., 2018). These effects appear to be mediated by CaMKII because cotreatment with heparinase I and the CaMKII inhibitor AIP fully rescues neuronal excitability and context discrimination and because the increase in CaMKII expression at the AIS is accompanied by changes in accumulation of ankyrin G. In summary, these data suggest that the CaMKII signaling cascade activated by ECM remodeling is essential for both HSP and the control of axonal excitability. So far, no specific GPCRs have been implicated in the mechanisms linking heparan sulfates to CaMKII. Still, heparan sulfates are known to modulate presentation of diverse positively charged ligands to GPCRs. For instance, they stabilize the formation of chemokine dimers and higher order chemokine oligomers that are required for binding to the G-protein-coupled chemokine receptors. One of these, CXCR4, is activated by chemokine C-X-C motif ligand CXCL12α bound to heparan sulfates (Thakar et al., 2017) and is known to regulate CaMKII activity (Hu et al., 2017).

## Homeostatic Plasticity and Metaplasticity

Synaptic and intrinsic homeostatic responses may cooperate with each other to maintain constant conditions for the induction of Hebbian-type synaptic plasticity. A good example is the aforementioned homeostatic regulation of HCN channels. Downscaling of excitatory synaptic currents following chronic network hyperactivity would favor subsequent induction of LTP because of the reduced initial synaptic strength. Concomitant homeostatic upregulation of HCN activity counteracts, however, the increased propensity of CA1 excitatory synapses to undergo LTP (Gasselin et al., 2015).

Similarly, downregulation of chondroitin sulfate-rich ECM increases signaling through β1 integrins, which may upregulate expression of GluN2B subunits of NMDARs (Schweitzer et al., 2017) and hence activates metaplastic mechanisms, which will promote synaptic plasticity (Song et al., 2019). These changes are counteracted by modulation of intrinsic excitability through activation of SK channels, which inhibits induction of LTP by theta-burst stimulation because of increased afterburst-hyperpolarization (Song et al., 2019).

Metaplasticity may occur also at the network level. For example K^+^ channels of the K_V_1 subfamily are enriched in the distal part of the AIS where they colocalize with scaffold proteins (PSD-93) and CAMs (contactin-associated protein-like 2, axonal glycoprotein TAG-1, and disintegrin and metalloproteinase domain-containing protein 22) (Leterrier, 2018). Although it is unclear whether these proteins are important for localizing K_V_1 channels at the AIS, high-frequency stimulation of the Schaffer collaterals has been shown to downregulate K_V_1 channel activity in hippocampal parvalbumin interneurons via activation of mGluR5. This enhances feed-forward inhibition mediated by parvalbumin interneurons, thus balancing increased synaptic and intrinsic excitation in CA1 pyramidal neurons (Campanac et al., 2013).

Another known ECM-dependent metaplastic mechanism is activated by deficiency in the ECM glycoprotein tenascin-R, which leads to upregulation of excitatory transmission to CA1 pyramidal neurons and reduction in perisomatic inhibition in the CA1 region through activation of postsynaptic metabotropic GABA_B_ receptors. This mechanism impairs TBS-LTP and results in a 10-mV metaplastic shift in the depolarization threshold necessary to induce LTP by low-frequency stimulation (Bukalo et al., 2007). In summary, downregulation of ECM may activate homeostatic non-Hebbian plasticity (via modulation of excitability) in parallel with metaplasticity (i.e., changes in rules of Hebbian plasticity) and call for careful dissection of their interplay in the context of neurological diseases.

## Concluding Remarks

**FIGURE 2:**
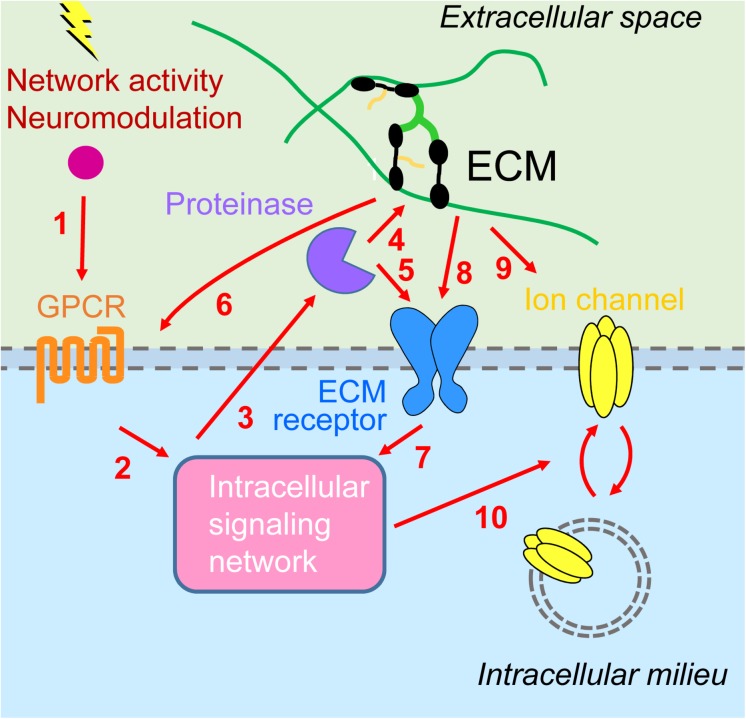
Interplay between metabotropic receptors and extracellular matrix in homeostatic plasticity. (1) Network activity and neuromodulatory systems stimulate G protein-coupled receptors (GPCRs) [metabotropic glutamate receptors 1/5 (mGluR1/5), D1/5 dopamine receptors, 5-HT7R serotonin receptors] and (2) downstream signaling networks [including calmodulin-dependent protein kinase II (CaMKII), protein kinase A (PKA), protein kinase C (PKC), and inositol trisphosphate receptors (IP_3_Rs)]. (3) This results in activation of extracellular proteinases [tumor necrosis factor-α-converting enzyme (TACE), disintegrin and metalloprotease with thrombospondin motifs 4/5 (ADAMTS4/5), matrix metalloproteinase 9 (MMP9)], which (4) may process ECM molecules (lecticans) or (5) ECM receptors (NPR, CD44), enabling synaptic modifications (not shown) as well as (6) signaling back through modulation of GPCRs (group III mGluR) and (7) additional intracellular signaling events. (8) Inactivity increases cell surface expression and signaling through major extracellular matrix (ECM) receptors, β3 integrins. (9) Activity stimulates secretion of Narp and its coaggregation with GluAs on interneurons. (10) Intracellular signaling cascades are converging on regulation of trafficking of GluAs [homeostatic synaptic plasticity (HSP)] or voltage- and Ca^2+^-gated ion channels (HIP).

We have highlighted emerging evidence suggesting a synergistic interplay between metabotropic receptors and ECM in regulating homeostatic plasticity. Activation of metabotropic receptors for glutamate, DA, and serotonin can initiate intracellular signaling pathways through tyrosine and serine kinases that culminate in the proteolytic cleavage of ECM molecules and ECM receptors. This structural remodeling of the extracellular environment provides either permissive or instructive conditions for HSP and HIP by regulating trafficking of synaptic and extrasynaptic ion channels, respectively. Furthermore, ECM proteins can also affect directly localization and signaling of metabotropic receptors. Although the experimental evidence is still scant, we propose that the superimposition of these reciprocal signaling pathways between intracellular and extracellular environments provides a robust and dynamic regulatory system for multiple forms of homeostatic plasticity (Figure 2).

## Author Contributions

LC, CV, and AD wrote the manuscript and designed figures.

## Conflict of Interest

The authors declare that the research was conducted in the absence of any commercial or financial relationships that could be construed as a potential conflict of interest.

## References

[B1] Adelman J. P. Maylie J. Sah P. (2012). Small-conductance Ca2+-activated K+ channels: form and function. *Annu. Rev. Physiol.* 74 245–269. 10.1146/annurev-physiol-020911-153336 21942705

[B2] Angelo K. London M. Christensen S. R. Hausser M. (2007). Local and global effects of I(h) distribution in dendrites of mammalian neurons. *J. Neurosci.* 27 8643–8653. 10.1523/jneurosci.5284-06.2007 17687042 PMC6672943

[B3] Ango F. Prezeau L. Muller T. Tu J. C. Xiao B. Worley P. F. (2001). Agonist-independent activation of metabotropic glutamate receptors by the intracellular protein Homer. *Nature* 411 962–965. 10.1038/35082096 11418862

[B4] Arimitsu T. Nuriya M. Ikeda K. Takahashi T. Yasui M. (2009). Activity-dependent regulation of HCN1 protein in cortical neurons. *Biochem. Biophys. Res. Commun.* 387 87–91. 10.1016/j.bbrc.2009.06.127 19563776

[B5] Arranz A. M. Perkins K. L. Irie F. Lewis D. P. Hrabe J. Xiao F. (2014). Hyaluronan deficiency due to Has3 knock-out causes altered neuronal activity and seizures via reduction in brain extracellular space. *J. Neurosci.* 34 6164–6176. 10.1523/JNEUROSCI.3458-13.2014 24790187 PMC4004806

[B6] Beffert U. Weeber E. J. Durudas A. Qiu S. Masiulis I. Sweatt J. D. (2005). Modulation of synaptic plasticity and memory by reelin involves differential splicing of the lipoprotein receptor Apoer2. *Neuron* 47 567–579. 10.1016/j.neuron.2005.07.007 16102539

[B7] Bianchi R. Chuang S. C. Zhao W. Young S. R. Wong R. K. (2009). Cellular plasticity for group I mGluR-mediated epileptogenesis. *J. Neurosci.* 29 3497–3507. 10.1523/JNEUROSCI.5447-08.2009 19295155 PMC2692254

[B8] Bianchi R. Wong R. K. S. Merlin L. R. (2012). “Glutamate receptors in epilepsy: group i mglur-mediated epileptogenesis,” in *Jasper’s Basic Mechanisms of the Epilepsies*, eds Noebels J. L. Avoli M. Rogawski M. A. Olsen R. W. Delgado-Escueta A. V. (Bethesda, MD: Oxford University Press). 10.1523/jneurosci.5447-08.2009 22787676

[B9] Biel M. Wahl-Schott C. Michalakis S. Zong X. (2009). Hyperpolarization-activated cation channels: from genes to function. *Physiol. Rev.* 89 847–885. 10.1152/physrev.00029.2008 19584315

[B10] Bijata M. Labus J. Guseva D. Stawarski M. Butzlaff M. Dzwonek J. (2017). Synaptic remodeling depends on signaling between serotonin receptors and the extracellular matrix. *Cell Rep.* 19 1767–1782. 10.1016/j.celrep.2017.05.023 28564597

[B11] Brager D. H. Johnston D. (2007). Plasticity of intrinsic excitability during long-term depression is mediated through mGluR-dependent changes in I(h) in hippocampal CA1 pyramidal neurons. *J. Neurosci.* 27 13926–13937. 10.1523/jneurosci.3520-07.2007 18094230 PMC6673524

[B12] Breton J. D. Stuart G. J. (2009). Loss of sensory input increases the intrinsic excitability of layer 5 pyramidal neurons in rat barrel cortex. *J. Physiol.* 587 5107–5119. 10.1113/jphysiol.2009.180943 19736297 PMC2790252

[B13] Bukalo O. Schachner M. Dityatev A. (2007). Hippocampal metaplasticity induced by deficiency in the extracellular matrix glycoprotein tenascin-R. *J. Neurosci.* 27 6019–6028. 10.1523/jneurosci.1022-07.2007 17537973 PMC6672247

[B14] Campanac E. Gasselin C. Baude A. Rama S. Ankri N. Debanne D. (2013). Enhanced intrinsic excitability in basket cells maintains excitatory-inhibitory balance in hippocampal circuits. *Neuron* 77 712–722. 10.1016/j.neuron.2012.12.020 23439123

[B15] Cannady R. McGonigal J. T. Newsom R. J. Woodward J. J. Mulholland P. J. Gass J. T. (2017). Prefrontal cortex KCa2 channels regulate mGlu5-dependent plasticity and extinction of alcohol-seeking behavior. *J. Neurosci.* 37 4359–4369. 10.1523/JNEUROSCI.2873-16.2017 28320841 PMC5413180

[B16] Cao Y. Sarria I. Fehlhaber K. E. Kamasawa N. Orlandi C. James K. N. (2015). Mechanism for selective synaptic wiring of rod photoreceptors into the retinal circuitry and its role in vision. *Neuron* 87 1248–1260. 10.1016/j.neuron.2015.09.002 26402607 PMC4583715

[B17] Chang M. C. Park J. M. Pelkey K. A. Grabenstatter H. L. Xu D. Linden D. J. (2010). Narp regulates homeostatic scaling of excitatory synapses on parvalbumin-expressing interneurons. *Nat. Neurosci.* 13 1090–1097. 10.1038/nn.2621 20729843 PMC2949072

[B18] Cho R. W. Park J. M. Wolff S. B. Xu D. Hopf C. Kim J. A. (2008). mGluR1/5-dependent long-term depression requires the regulated ectodomain cleavage of neuronal pentraxin NPR by TACE. *Neuron* 57 858–871. 10.1016/j.neuron.2008.01.010 18367087 PMC2701195

[B19] Cingolani L. A. Thalhammer A. Yu L. M. Catalano M. Ramos T. Colicos M. A. (2008). Activity-dependent regulation of synaptic AMPA receptor composition and abundance by beta3 integrins. *Neuron* 58 749–762. 10.1016/j.neuron.2008.04.011 18549786 PMC2446609

[B20] de Wit J. Ghosh A. (2016). Specification of synaptic connectivity by cell surface interactions. *Nat. Rev. Neurosci.* 17 22–35. 10.1038/nrn.2015.3 26656254

[B21] Debanne D. Inglebert Y. Russier M. (2019). Plasticity of intrinsic neuronal excitability. *Curr. Opin. Neurobiol.* 54 73–82. 10.1016/j.conb.2018.09.001 30243042

[B22] Desai N. S. Rutherford L. C. Turrigiano G. G. (1999). Plasticity in the intrinsic excitability of cortical pyramidal neurons. *Nat. Neurosci.* 2 515–520. 10.1038/9165 10448215

[B23] Diering G. H. Nirujogi R. S. Roth R. H. Worley P. F. Pandey A. Huganir R. L. (2017). Homer1a drives homeostatic scaling-down of excitatory synapses during sleep. *Science* 355 511–515. 10.1126/science.aai8355 28154077 PMC5382711

[B24] Dolan J. Mitchell K. J. (2013). Mutation of Elfn1 in mice causes seizures and hyperactivity. *PLoS One* 8:e80491. 10.1371/journal.pone.0080491 24312227 PMC3842350

[B25] Dunn H. A. Patil D. N. Cao Y. Orlandi C. Martemyanov K. A. (2018). Synaptic adhesion protein ELFN1 is a selective allosteric modulator of group III metabotropic glutamate receptors in trans. *Proc. Natl. Acad. Sci. U.S.A.* 115 5022–5027. 10.1073/pnas.1722498115 29686062 PMC5948991

[B26] El-Hassar L. Hagenston A. M. D’Angelo L. B. Yeckel M. F. (2011). Metabotropic glutamate receptors regulate hippocampal CA1 pyramidal neuron excitability via Ca(2)(+) wave-dependent activation of SK and TRPC channels. *J. Physiol.* 589 3211–3229. 10.1113/jphysiol.2011.209783 21576272 PMC3145935

[B27] Ferrer-Ferrer M. Dityatev A. (2018). Shaping synapses by the neural extracellular matrix. *Front. Neuroanat.* 12:40. 10.3389/fnana.2018.00040 29867379 PMC5962695

[B28] Garcia-Negredo G. Soto D. Llorente J. Morato X. Galenkamp K. M. Gomez-Soler M. (2014). Coassembly and coupling of SK2 channels and mGlu5 receptors. *J. Neurosci.* 34 14793–14802. 10.1523/JNEUROSCI.2038-14.2014 25355231 PMC6608419

[B29] Gasselin C. Inglebert Y. Debanne D. (2015). Homeostatic regulation of h-conductance controls intrinsic excitability and stabilizes the threshold for synaptic modification in CA1 neurons. *J. Physiol.* 593 4855–4869. 10.1113/JP271369 26316265 PMC4650411

[B30] Gladding C. M. Collett V. J. Jia Z. Bashir Z. I. Collingridge G. L. Molnar E. (2009). Tyrosine dephosphorylation regulates AMPAR internalisation in mGluR-LTD. *Mol. Cell Neurosci.* 40 267–279. 10.1016/j.mcn.2008.10.014 19063969

[B31] Gymnopoulos M. Cingolani L. A. Pedarzani P. Stocker M. (2014). Developmental mapping of small-conductance calcium-activated potassium channel expression in the rat nervous system. *J. Comp. Neurol.* 522 1072–1101. 10.1002/cne.23466 24096910 PMC4016743

[B32] Hagenston A. M. Fitzpatrick J. S. Yeckel M. F. (2008). MGluR-mediated calcium waves that invade the soma regulate firing in layer V medial prefrontal cortical pyramidal neurons. *Cereb. Cortex* 18 407–423. 10.1093/cercor/bhm075 17573372 PMC3005283

[B33] Hahn G. Ponce-Alvarez A. Deco G. Aertsen A. Kumar A. (2019). Portraits of communication in neuronal networks. *Nat. Rev. Neurosci.* 20 117–127. 10.1038/s41583-018-0094-0 30552403

[B34] Hengen K. B. Torrado Pacheco A. McGregor J. N. Van Hooser S. D. Turrigiano G. G. (2016). Neuronal firing rate homeostasis is inhibited by sleep and promoted by wake. *Cell* 165 180–191. 10.1016/j.cell.2016.01.046 26997481 PMC4809041

[B35] Hu J. H. Park J. M. Park S. Xiao B. Dehoff M. H. Kim S. (2010). Homeostatic scaling requires group I mGluR activation mediated by homer1a. *Neuron* 68 1128–1142. 10.1016/j.neuron.2010.11.008 21172614 PMC3013614

[B36] Hu X. M. Zhang H. Xu H. Zhang H. L. Chen L. P. Cui W. Q. (2017). Chemokine receptor CXCR4 regulates CaMKII/CREB pathway in spinal neurons that underlies cancer-induced bone pain. *Sci. Rep.* 7:4005. 10.1038/s41598-017-04198-3 28638088 PMC5479786

[B37] Ito M. Nagai T. Mizoguchi H. Sato K. Hayase M. Otsuka N. (2007). Activation of post-synaptic dopamine D(1) receptors promotes the release of tissue plasminogen activator in the nucleus accumbens via PKA signaling. *J. Neurochem.* 103 2589–2596. 10.1111/j.1471-4159.2007.04946.x 17944865

[B38] Jang S. S. Royston S. E. Xu J. Cavaretta J. P. Vest M. O. Lee K. Y. (2015). Regulation of STEP61 and tyrosine-phosphorylation of NMDA and AMPA receptors during homeostatic synaptic plasticity. *Mol. Brain* 8:55. 10.1186/s13041-015-0148-4 26391783 PMC4578242

[B39] Jaudon F. Thalhammer A. Cingolani L. A. (2019). Correction of β3 integrin haplo-insufficiency by CRISPRa normalizes cortical network activity. *bioRxiv* [Preprint]. 10.1101/664706

[B40] Jeanneret V. Yepes M. (2017). Tissue-type plasminogen activator is a homeostatic regulator of synaptic function in the central nervous system. *Neural Regen. Res.* 12 362–365. 10.4103/1673-5374.202924 28469640 PMC5399703

[B41] Kaneko M. Stellwagen D. Malenka R. C. Stryker M. P. (2008). Tumor necrosis factor-alpha mediates one component of competitive, experience-dependent plasticity in developing visual cortex. *Neuron* 58 673–680. 10.1016/j.neuron.2008.04.023 18549780 PMC2884387

[B42] Kirchheim F. Tinnes S. Haas C. A. Stegen M. Wolfart J. (2013). Regulation of action potential delays via voltage-gated potassium Kv1.1 channels in dentate granule cells during hippocampal epilepsy. *Front. Cell Neurosci.* 7:248. 10.3389/fncel.2013.00248 24367293 PMC3852106

[B43] Kochlamazashvili G. Henneberger C. Bukalo O. Dvoretskova E. Senkov O. Lievens P. M. (2010). The extracellular matrix molecule hyaluronic acid regulates hippocampal synaptic plasticity by modulating postsynaptic L-type Ca(2+) channels. *Neuron* 67 116–128. 10.1016/j.neuron.2010.05.030 20624596 PMC3378029

[B44] Korotchenko S. Cingolani L. A. Kuznetsova T. Bologna L. L. Chiappalone M. Dityatev A. (2014). Modulation of network activity and induction of homeostatic synaptic plasticity by enzymatic removal of heparan sulfates. *Philos. Trans. R. Soc. Lond. B Biol. Sci.* 369:20140134. 10.1098/rstb.2014.0134 25225107 PMC4173299

[B45] Kuba H. Yamada R. Ishiguro G. Adachi R. (2015). Redistribution of Kv1 and Kv7 enhances neuronal excitability during structural axon initial segment plasticity. *Nat. Commun.* 6:8815. 10.1038/ncomms9815 26581625 PMC4673506

[B46] Kupferman J. V. Basu J. Russo M. J. Guevarra J. Cheung S. K. Siegelbaum S. A. (2014). Reelin signaling specifies the molecular identity of the pyramidal neuron distal dendritic compartment. *Cell* 158 1335–1347. 10.1016/j.cell.2014.07.035 25201528 PMC4183142

[B47] Lemarchant S. Pruvost M. Hebert M. Gauberti M. Hommet Y. Briens A. (2014). tPA promotes ADAMTS-4-induced CSPG degradation, thereby enhancing neuroplasticity following spinal cord injury. *Neurobiol. Dis.* 66 28–42. 10.1016/j.nbd.2014.02.005 24576594

[B48] Leterrier C. (2018). The axon initial segment: an updated viewpoint. *J. Neurosci.* 38 2135–2145. 10.1523/JNEUROSCI.1922-17.2018 29378864 PMC6596274

[B49] Lujan R. Aguado C. Ciruela F. Arus X. M. Martin-Belmonte A. Alfaro-Ruiz R. (2018). SK2 channels associate with mglu1alpha receptors and cav2.1 channels in purkinje cells. *Front. Cell Neurosci.* 12:311. 10.3389/fncel.2018.00311 30283304 PMC6156379

[B50] Lujan R. Maylie J. Adelman J. P. (2009). New sites of action for GIRK and SK channels. *Nat. Rev. Neurosci.* 10 475–480. 10.1038/nrn2668 19543219

[B51] Luscher C. Huber K. M. (2010). Group 1 mGluR-dependent synaptic long-term depression: mechanisms and implications for circuitry and disease. *Neuron* 65 445–459. 10.1016/j.neuron.2010.01.016 20188650 PMC2841961

[B52] Maffei A. Turrigiano G. G. (2008). Multiple modes of network homeostasis in visual cortical layer 2/3. *J. Neurosci.* 28 4377–4384. 10.1523/JNEUROSCI.5298-07.2008 18434516 PMC2655203

[B53] Maiza A. Chantepie S. Vera C. Fifre A. Huynh M. B. Stettler O. (2018). The role of heparan sulfates in protein aggregation and their potential impact on neurodegeneration. *FEBS Lett.* 592 3806–3818. 10.1002/1873-3468.13082 29729013

[B54] McGeachie A. B. Skrzypiec A. E. Cingolani L. A. Letellier M. Pawlak R. Goda Y. (2012). beta3 integrin is dispensable for conditioned fear and Hebbian forms of plasticity in the hippocampus. *Eur. J. Neurosci.* 36 2461–2469. 10.1111/j.1460-9568.2012.08163.x 22748100 PMC5122446

[B55] Megias M. Emri Z. Freund T. F. Gulyas A. I. (2001). Total number and distribution of inhibitory and excitatory synapses on hippocampal CA1 pyramidal cells. *Neuroscience* 102 527–540. 10.1016/s0306-4522(00)00496-6 11226691

[B56] Milshtein-Parush H. Frere S. Regev L. Lahav C. Benbenishty A. Ben-Eliyahu S. (2017). Sensory deprivation triggers synaptic and intrinsic plasticity in the hippocampus. *Cereb. Cortex* 27 3457–3470. 10.1093/cercor/bhx084 28407141

[B57] Minge D. Senkov O. Kaushik R. Herde M. K. Tikhobrazova O. Wulff A. B. (2017). Heparan sulfates support pyramidal cell excitability, synaptic plasticity, and context discrimination. *Cereb. Cortex* 27 903–918. 10.1093/cercor/bhx003 28119345 PMC5390399

[B58] Mironov A. Song I. Senkov O. Kuznetsova T. Hayani H. Druzin M. (2018). *A CaMKII-Dependent Mechanism Underlying Impaired Neuronal Excitability and Contextual Discrimination After Enzymatic Removal of Heparan Sulfates in the CA1 Region of Mouse Hippocampus.* Berlin: FENS Forum.

[B59] Mitlöhner J. Kaushik R. Niekisch H. Blondiaux A. Gee C. E. Happel M. F. K. (2019). Dopamine modulates the integrity of the perisynaptic extracellular matrix at excitatory synapses. *bioRxiv* [Preprint] 10.1101/722454 PMC7073179 31972963

[B60] Moult P. R. Gladding C. M. Sanderson T. M. Fitzjohn S. M. Bashir Z. I. Molnar E. (2006). Tyrosine phosphatases regulate AMPA receptor trafficking during metabotropic glutamate receptor-mediated long-term depression. *J. Neurosci.* 26 2544–2554. 10.1523/jneurosci.4322-05.2006 16510732 PMC6793648

[B61] Niswender C. M. Conn P. J. (2010). Metabotropic glutamate receptors: physiology, pharmacology, and disease. *Annu. Rev. Pharmacol. Toxicol.* 50 295–322. 10.1146/annurev.pharmtox.011008.145533 20055706 PMC2904507

[B62] O’Connor E. C. Bariselli S. Bellone C. (2014). Synaptic basis of social dysfunction: a focus on postsynaptic proteins linking group-I mGluRs with AMPARs and NMDARs. *Eur. J. Neurosci.* 39 1114–1129. 10.1111/ejn.12510 24712991

[B63] Oliet S. H. Malenka R. C. Nicoll R. A. (1997). Two distinct forms of long-term depression coexist in CA1 hippocampal pyramidal cells. *Neuron* 18 969–982. 10.1016/s0896-6273(00)80336-0 9208864

[B64] Pedarzani P. Stocker M. (2008). Molecular and cellular basis of small- and intermediate-conductance, calcium-activated potassium channel function in the brain. *Cell. Mol. Life Sci.* 65 3196–3217. 10.1007/s00018-008-8216-x 18597044 PMC2798969

[B65] Pin J. P. Bettler B. (2016). Organization and functions of mGlu and GABAB receptor complexes. *Nature* 540 60–68. 10.1038/nature20566 27905440

[B66] Piskorowski R. Santoro B. Siegelbaum S. A. (2011). TRIP8b splice forms act in concert to regulate the localization and expression of HCN1 channels in CA1 pyramidal neurons. *Neuron* 70 495–509. 10.1016/j.neuron.2011.03.023 21555075 PMC3107038

[B67] Pozo K. Cingolani L. A. Bassani S. Laurent F. Passafaro M. Goda Y. (2012). beta3 integrin interacts directly with GluA2 AMPA receptor subunit and regulates AMPA receptor expression in hippocampal neurons. *Proc. Natl. Acad. Sci. U.S.A.* 109 1323–1328. 10.1073/pnas.1113736109 22232691 PMC3268285

[B68] Sansig G. Bushell T. J. Clarke V. R. Rozov A. Burnashev N. Portet C. (2001). Increased seizure susceptibility in mice lacking metabotropic glutamate receptor 7. *J. Neurosci.* 21 8734–8745. 10.1523/jneurosci.21-22-08734.2001 11698585 PMC6762269

[B69] Schanzenbacher C. T. Langer J. D. Schuman E. M. (2018). Time- and polarity-dependent proteomic changes associated with homeostatic scaling at central synapses. *eLife* 7 e33322. 10.7554/eLife.33322 29447110 PMC5814146

[B70] Scholz R. Berberich S. Rathgeber L. Kolleker A. Kohr G. Kornau H. C. (2010). AMPA receptor signaling through BRAG2 and Arf6 critical for long-term synaptic depression. *Neuron* 66 768–780. 10.1016/j.neuron.2010.05.003 20547133

[B71] Schweitzer B. Singh J. Fejtova A. Groc L. Heine M. Frischknecht R. (2017). Hyaluronic acid based extracellular matrix regulates surface expression of GluN2B containing NMDA receptors. *Sci. Rep.* 7:10991. 10.1038/s41598-017-07003-3 28887453 PMC5591221

[B72] Shim H. G. Jang S. S. Jang D. C. Jin Y. Chang W. Park J. M. (2016). mGlu1 receptor mediates homeostatic control of intrinsic excitability through Ih in cerebellar Purkinje cells. *J. Neurophysiol.* 115 2446–2455. 10.1152/jn.00566.2015 26912592 PMC4922465

[B73] Shin M. Chetkovich D. M. (2007). Activity-dependent regulation of h channel distribution in hippocampal CA1 pyramidal neurons. *J. Biol. Chem.* 282 33168–33180. 10.1074/jbc.m703736200 17848552 PMC2685032

[B74] Silberberg G. Grillner S. LeBeau F. E. Maex R. Markram H. (2005). Synaptic pathways in neural microcircuits. *Trends Neurosci.* 28 541–551. 10.1016/j.tins.2005.08.004 16122815

[B75] Song I. Singh J. Wirth A. Minge D. Kaushik R. Ferrer-Ferrer M. (2019). *Extracellular Matrix Balances Principal Cell Excitability and Synaptic Plasticity. Program No. 463.10.2019 Neuroscience Meeting Planner, Chicago.* Available at: https://www.abstractsonline.com/pp8/#!/7883/presentation/61569

[B76] Sourdet V. Russier M. Daoudal G. Ankri N. Debanne D. (2003). Long-term enhancement of neuronal excitability and temporal fidelity mediated by metabotropic glutamate receptor subtype 5. *J. Neurosci.* 23 10238–10248. 10.1523/jneurosci.23-32-10238.2003 14614082 PMC6741009

[B77] Stachniak T. J. Sylwestrak E. L. Scheiffele P. Hall B. J. Ghosh A. (2019). Elfn1-Induced constitutive activation of mGluR7 determines frequency-dependent recruitment of *Somatostatin interneurons*. *J. Neurosci.* 39 4461–4474. 10.1523/JNEUROSCI.2276-18.2019 30940718 PMC6554623

[B78] Stellwagen D. Malenka R. C. (2006). Synaptic scaling mediated by glial TNF-alpha. *Nature* 440 1054–1059. 10.1038/nature04671 16547515

[B79] Stocker M. Pedarzani P. (2000). Differential distribution of three Ca(2+)-activated K(+) channel subunits, SK1, SK2, and SK3, in the adult rat central nervous system. *Mol. Cell Neurosci.* 15 476–493. 10.1006/mcne.2000.0842 10833304

[B80] Stuart G. J. Spruston N. (2015). Dendritic integration: 60 years of progress. *Nat. Neurosci.* 18 1713–1721. 10.1038/nn.4157 26605882

[B81] Sylwestrak E. L. Ghosh A. (2012). Elfn1 regulates target-specific release probability at CA1-interneuron synapses. *Science* 338 536–540. 10.1126/science.1222482 23042292 PMC5297939

[B82] Thakar D. Dalonneau F. Migliorini E. Lortat-Jacob H. Boturyn D. Albiges-Rizo C. (2017). Binding of the chemokine CXCL12alpha to its natural extracellular matrix ligand heparan sulfate enables myoblast adhesion and facilitates cell motility. *Biomaterials* 123 24–38. 10.1016/j.biomaterials.2017.01.022 28152381 PMC5405871

[B83] Thiagarajan T. C. Lindskog M. Tsien R. W. (2005). Adaptation to synaptic inactivity in hippocampal neurons. *Neuron* 47 725–737. 10.1016/j.neuron.2005.06.037 16129401

[B84] Tomioka N. H. Yasuda H. Miyamoto H. Hatayama M. Morimura N. Matsumoto Y. (2014). Elfn1 recruits presynaptic mGluR7 in trans and its loss results in seizures. *Nat. Commun.* 5:4501. 10.1038/ncomms5501 25047565

[B85] Tononi G. Cirelli C. (2014). Sleep and the price of plasticity: from synaptic and cellular homeostasis to memory consolidation and integration. *Neuron* 81 12–34. 10.1016/j.neuron.2013.12.025 24411729 PMC3921176

[B86] Valenzuela J. C. Heise C. Franken G. Singh J. Schweitzer B. Seidenbecher C. I. (2014). Hyaluronan-based extracellular matrix under conditions of homeostatic plasticity. *Philos. Trans. R. Soc. Lond. B Biol. Sci.* 369:20130606. 10.1098/rstb.2013.0606 25225099 PMC4173291

[B87] Vedunova M. Sakharnova T. Mitroshina E. Perminova M. Pimashkin A. Zakharov Y. (2013). Seizure-like activity in hyaluronidase-treated dissociated hippocampal cultures. *Front. Cell Neurosci.* 7:149. 10.3389/fncel.2013.00149 24062641 PMC3770920

[B88] Wang Y. Fehlhaber K. E. Sarria I. Cao Y. Ingram N. T. Guerrero-Given D. (2017). The auxiliary calcium channel subunit alpha2delta4 Is required for axonal elaboration, synaptic transmission, and wiring of rod photoreceptors. *Neuron* 93 1359–1374. 10.1016/j.neuron.2017.02.021 28262416 PMC5364038

[B89] Xu J. Y. Chen R. Zhang J. Chen C. (2010). Endocannabinoids differentially modulate synaptic plasticity in rat hippocampal CA1 pyramidal neurons. *PLoS One* 5:e10306. 10.1371/journal.pone.0010306 20421986 PMC2858667

[B90] Young S. R. Chuang S. C. Zhao W. Wong R. K. Bianchi R. (2013). Persistent receptor activity underlies group I mGluR-mediated cellular plasticity in CA3 neuron. *J. Neurosci.* 33 2526–2540. 10.1523/JNEUROSCI.3338-12.2013 23392681 PMC3711659

